# Type IX Collagen Turnover Is Altered in Patients with Solid Tumors

**DOI:** 10.3390/cancers16112035

**Published:** 2024-05-27

**Authors:** Helena Port, Yi He, Morten A. Karsdal, Emilie A. Madsen, Anne-Christine Bay-Jensen, Nicholas Willumsen, Signe Holm Nielsen

**Affiliations:** 1 Immunoscience, Nordic Bioscience, 2730 Herlev, Denmark; yhe@nordicbio.com (Y.H.); mk@nordicbio.com (M.A.K.); eam@nordicbio.com (E.A.M.); acbj@nordicbio.com (A.-C.B.-J.); nwi@nordicbio.com (N.W.); shn@nordicbio.com (S.H.N.); 2 Department of Biomedical Sciences, University of Copenhagen, 2200 Copenhagen, Denmark

**Keywords:** type IX collagen, FACIT, extracellular matrix, non-invasive biomarker, epitope, tissue turnover

## Abstract

**Simple Summary:**

The extracellular matrix, composed of various collagens including the fibril-associated collagens with interrupted triple helices (FACIT), plays a crucial role in cancer development and progression. Type IX collagen belongs to the FACIT family, yet its specific role in cancer biology remains unclear. Understanding the impact of collagen type IX in the tumor microenvironment can provide insights into how changes in the ECM contribute to cancer development and metastasis. By identifying biomarkers derived from type IX collagen turnover that reflect ECM alterations, we can quantify these changes and better understand cancer pathogenesis. This article introduces a type IX collagen biomarker that could serve as a diagnostic tool for various types of solid tumors.

**Abstract:**

The fibrotic tumor microenvironment, characterized by its intricate extracellular matrix (ECM), consists of many collagens with diverse functions and unexplored biomarker potential. Type IX collagen is a member of the low-abundance collagen family known as the fibril-associated collagen with interrupted triple helices (FACITs) and is found mostly in cartilage. Its role in the tumor microenvironment remains unexplored. To investigate the biomarker potential of a type IX collagen in cancer, an immuno-assay was developed (PRO-C9) and technical assay performance was evaluated for the assessment of serum. PRO-C9 levels were measured in serum samples from 259 patients with various solid tumor types compared to serum levels from 73 healthy controls. PRO-C9 levels were significantly elevated in patients with solid tumors including bladder, breast, colorectal, gastric, head and neck, lung, melanoma, ovarian, pancreatic, and renal compared to levels in healthy controls (*p* < 0.05–*p* < 0.0001). PRO-C9 could discriminate between patients with cancer and healthy controls, with the area under the receiver operating characteristic values ranging from 0.58 to 0.86 (*p* < 0.3–*p* < 0.0001), indicating potential diagnostic utility. This study suggests that type IX collagen turnover is altered in patients with solid tumors and demonstrates the feasibility of using PRO-C9 as a non-invasive serum-based biomarker with relevance in multiple cancer types. Furthermore, these results underscore the potential utility of PRO-C9 to better elucidate the biology of FACITs in cancers.

## 1. Introduction

The extracellular matrix (ECM) is the non-cellular part of tissues, playing a vital role in tissue and organ structure as well as cellular polarity and activity [1]. In healthy tissue, ECM homeostasis is kept intact by the ongoing degradation of old proteins and the synthesis of new proteins. In cancer, ECM homeostasis is lost due to excessive remodeling of the ECM consequent to the overactivation of fibroblasts, chronic inflammatory processes, and tumor cell activity [2]. Ultimately, this leads to a pro-tumorigenic microenvironment, which is a hallmarks of cancer [3,4,5]. Understanding changes in the composition and quality of the tumor microenvironment (TME) is therefore key in cancer research and treatment. 

Collagens are the most prominent ECM proteins in solid tumors, of which there are 28 different types with 46 side chains [6]. Traditionally, in cancer research, the relatively abundant collagens such as type I, III and IV collagen have been extensively studied and are known to be associated with tumor fibrosis, invasion, metastasis, and angiogenesis [7,8]. Inflammatory responses within the tumor microenvironment drive extensive ECM remodeling, characterized by the turnover of collagens and subsequent replacement of new proteins as tumors evolve [9]. The resulting release of collagen fragments can serve as a source of non-invasive biomarkers for several types of cancer, with findings suggesting an association with disease activity and prognosis [10,11,12,13,14,15,16,17]. 

Recently, a subclass of low-abundance collagens known as the fibril-associated collagen with interrupted triple helices (FACITs) has been proposed as non-invasive biomarkers for cancer [18,19,20,21]. Type IX collagen belongs to the FACIT family, and each of the type IX molecules consists of three α-chains (α-chain 1, α-chain 2 and α-chain 3) that are interspersed and flanked by non-collagenous domains [22]. Biomarkers derived from FACIT collagens such as type XVI, XIX, XX and XXII have already been developed, and their serum levels have been observed to rise in sera from patients with cancer and are associated with poor overall survival [18,19,20,21]. Other FACIT collagens such as type XIV and XIX collagen have also shown relevance in cancer. Proteomic investigations of the matrisome of metastatic colon cancers have revealed a heightened presence of type XIV collagen peptides compared to healthy human colons [23]. It has also been found that type XIX collagen is implicated in cancer progression. Amenta et al. [24] found that collagen type XIX is lost in the development of invasive tumors in the basement membrane zone, suggesting that its loss might signal the remodeling of the ECM to promote tumor cell infiltration. In addition, Ramont et al. [25] found that the NC1 domain of type XIX collagen inhibits melanoma cell migration, suggesting its role in antitumor and antiangiogenic activity. 

Mechanistically, while type I, III and IV collagen are fibrillar and network-forming collagens and therefore make up the bulk of the collagenous ECM, FACITs are thought to play a delicate and unique role by forming molecular bridges, and thereby facilitate the stability of the collagenous network in the ECM [26,27]. Thus, the altered FACIT remodeling seen in cancer patients may be indicative of the loss of tissue and ECM architecture. Even though type IX collagen is known to be located most abundantly in cartilage [27], we hypothesize that it might possess serum biomarker potential in cancer, as it might be implicated in creating a supportive niche for cancer cells within the TME. In the present study, we developed an immunoassay targeting the C-terminal domain of the α-chain 1 of type IX collagen (PRO-C9) in serum and evaluated the biomarker potential in a cohort of patients with various types of cancer by comparing PRO-C9 levels across tumor types and relative to healthy controls. 

## 2. Materials and Methods

All reagents used were chemicals from Merck (Whitehouse Station, NJ, USA) and Sigma (St. Louis, MO, USA) unless stated otherwise. All synthetic peptides used for antibody production and assay validation were purchased from Genscript (Piscataway, NJ, USA) (Table 1).

### 2.1. Monoclonal Antibody Development, Production, and Characterization

The amino acid sequence ^912^′QRAFNKGPDP′^921^ was used as the immunogen for raising the monoclonal antibodies. Immunization was initiated by the subcutaneous injection of 200 μL emulsified antigen and 100 μg immunogenic peptide (KLH-CGG-QRAFNKGPDP) in 4- to 6-week-old Balb/C mice using Stimmune (Thermo Fisher, Waltham, MA, USA). The immunizations were repeated every second week until stable serum antibody titer levels were reached. The mouse with the highest serum titer was selected for fusion and rested for a month. Subsequently, the mouse was boosted intravenously with 50 μg immunogenic peptide in 100 μL 0.9% NaCl solution 3 days before isolation of the spleen for cell fusion. To produce hybridoma cells, the mouse spleen cells were fused with SP2/0 myeloma cells as described by Gefter et al. [28]. Subsequently, the clones were plated into 96-well microtiter plates for further growth, and the limiting dilution method was applied to promote monoclonal growth. A competitive ELISA performed on streptavidin-coated plates was used for the screening of supernatant reactivity. Biotin-QRAFNKGPDP was used as the screening peptide, while the standard peptide QRAFNKGPDP was used to further test the specificity of the clones. Antibody specificity was calculated as a percentage of signal inhibition by two-fold-diluted standard peptide (QRAFNKGPDP), elongated peptide (QRAFNKGPDPG), truncated peptide (QRAFNKGPD), and non-sense peptide (DQAAGGLRQH). Supernatant was collected from the hybridoma cells and purified using HiTrap affinity columns (GEHealthcare Life Science, Little Chalfont, Buckinghamshire, UK) according to the manufacturer’s instructions. Antibody isotype was determined using the Rapid ELISA Mouse Monoclonal Antibody Isotyping Kit (Invitro-gen, Carlsbad, CA, USA) following the manufacturer’s specified protocol.

Native reactivity was assessed using human serum purchased from a commercial supplier (Valley Biomedical, Winchester, VA, USA).

### 2.2. PRO-C9 Assay Development

Development of the PRO-C9 competitive chemiluminescence immunoassay (CLIA) included several preliminary optimizing experiments where reagents, concentrations, incubation time and temperature were analyzed by several tests. The final CLIA procedure is as follows: A 96-well streptavidin-coated white microplate (Greiner Bio-One, Kremsmünster, Austria) was coated with 5 ng/mL biotinylated synthetic peptide (Biotin-QRAFNKGPDP) dissolved in assay buffer (10 mM phosphate buffered saline (PBS), 1% bovine serum albumin, 0.1% Tween-20, 0.36% Bronidox, 4 g/L NaCl, adjusted to pH 7.4 at 20 °C) and incubated for 30 min at 20 °C with constant shaking (300 rpm) in darkness. Next, 20 µL/well of standard peptide (240 ng/mL) and samples were added to the appropriate wells, followed by the addition of 100 μL/well of HRP-labeled antibody diluted in assay buffer to the concertation of 100 ng/mL and incubated for 20 h at 4 °C with constant shaking (300 rpm) in darkness. After each incubation step, the wells were washed with standard washing buffer (20 mM Tris, 50 mM NaCl, pH 7.2). The chemiluminescence substrate (Roche, BM Chemiluminescence ELISA substrate (POD), Basel, Switzerland) working solutions were mixed 15 min before use and 100 μL/well were added to the plate and incubated for 3 min at 20 °C with constant shaking (300 rpm) in darkness. The relative light units were measured at all wavelengths within 5 min on a microplate luminometer reader (SpectraMax M5, Molecular Devices, CA, USA). A standard curve was plotted using a 4-parameter logistic curve fit Y = (A − D)/(1 + (x/C)^B) + D, where R > 0.9. Data were analyzed using the SoftMax Pro version 7.0.3 software.

### 2.3. Technical Evaluation

Linearity was assessed using four human serum samples, diluted from two-fold down to an eight-fold dilution, and was calculated as a percentage of recovery from the diluted sample with an acceptance criterion of 80–120%. The intra- and inter-assay variation was determined by 10 independent runs of five serum samples and two kit controls run in double determinations. The variation range was based on the coefficient of variance (CV)% between the 10 plates and the criteria of acceptance was mean CV% ≤ 20%. Analytical interference was performed by adding a low/high content of hemoglobin (2.50/5 mg/mL), lipemia/lipids (1.50/5 mg/mL) and biotin (3/9 ng/mL) to a serum sample of known concentration. The normal reference levels for hemoglobin, lipidemia/lipids and biotin are 0–10 mg/dL (0–0.00161 mmol/L), <150 mg/dL (<1.6935 mmol/L) and 0.221–3.004 ng/mL, respectively. The percentage recovery of the analyte for each interference sample was calculated using the non-spiked serum as a reference, with an acceptance criterion of 80–120%. The measurement range was defined as the range between the lower limit of quantification range (LLOQ) and the upper limit of quantification range (ULOQ). The LLOQ was determined by measuring four low-level human serum samples in triplicate across five separate runs. The ULOQ was defined as the highest standard point on the assay’s standard curve from 10 independent runs that met the criteria for acceptable accuracy and precision. The standard curve robustness and the half-maximal inhibition concentration (IC50) were also determined. The analyte stability was examined through temperature tests and repeated freeze–thaw cycles of serum samples. The temperature tests included different time points and temperatures where PRO-C9 levels were measured in three human serum samples after 0, 2, 4, 24, and 24 h of incubation at either 4 °C or 20 °C. The recovery was calculated based on the relative error (RE) % relative to the samples that underwent a single thawing prior to the analysis with an acceptance criterion within 80–120%. Furthermore, the effect of four repeated freeze–thaw cycles of three serum samples was assessed, where freeze–thaw recovery was calculated with the zero cycle samples as a reference. Each sample was run in double determination.

### 2.4. Biological Evaluation of PRO-C9

The biological utility of PRO-C9 was evaluated in serum samples from 11 groups of patients with different solid tumor types (bladder, breast, colorectal, gastric, head and neck, lung, melanoma, ovarian, pancreatic, prostate, or renal). The levels of PRO-C9 in the solid tumor types were compared to the levels of self-reported healthy donors. The serum samples were acquired from the commercial vendor Proteogenex (Culver City, CA, USA) and BioIVT (Westbury, NY, USA). Serum samples were obtained and stored at −80 °C until analysis. 

### 2.5. Statistical Analysis

The biomarker data were considered non-parametric after a visual assessment of normality using density plots. The statistical comparisons of PRO-C9 between the 11 solid tumor types and healthy donors were performed by a Kruskal–Wallis test with Dunn’s multiple comparison test. Diagnostic accuracy was tested by AUROC. A *p*-value below 0.05 was considered significant. Statistical analyses and graphs were developed using GraphPad Prism version 9 (GraphPad Software, Inc., La Jolla, CA, USA) and R studio version 4.2.1 (R Foundation for Statistical Computing, Vienna, Austria.).

## 3. Results

### 3.1. Technical Evaluation and Characterization of PRO-C9

**Figure 1:**
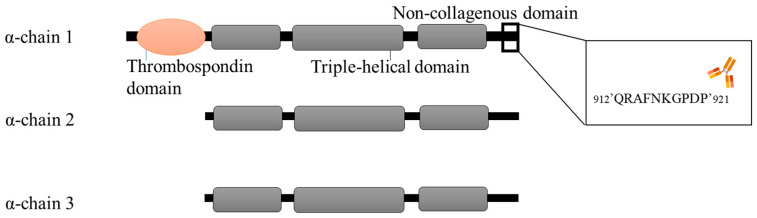
Primary structure of type IX collagen, indicating the PRO-C9 epitope location. Figure adapted from Karsdal et al. [1].

**Figure 2:**
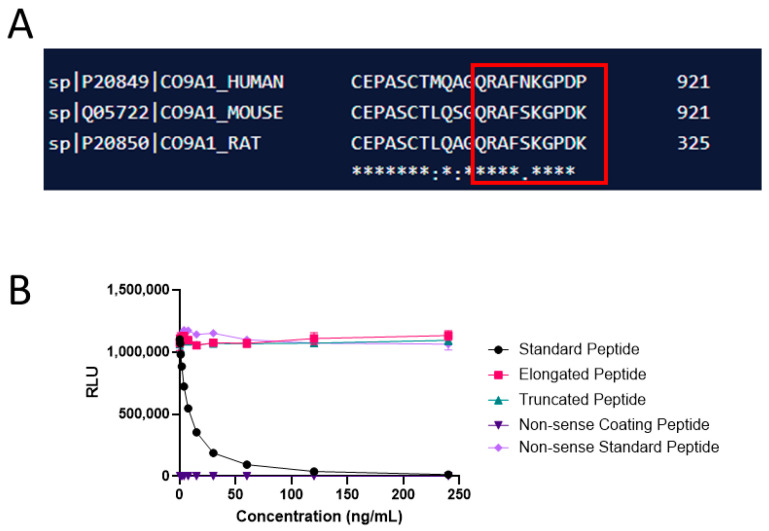
Alignment and specificity of the PRO-C9 assay. (**A**) Sequence alignment of the targeted sequence for PRO-C9 in human with mouse and rat. Sequence is marked with a red box. Asterisks indicate the amino acids match in all sequences, while dots indicate mismatches. (**B**) Specificity of the PRO-C9 assay. Reactivity towards the standard peptide (QRAFNKGPDP), truncated peptide (QRAFNKGPD), elongated peptide (QRAFNKGPDPG) and non-sense standard peptide and coater (DQAAGGLRQH). Signals are shown as relative luminescence (RLU) per second, as a function of standard peptide.

A schematic representation of the PRO-C9 epitope location can be found in Figure 1, while a summary of the technical evaluation of the PRO-C9 assay can be found in Table 2. Briefly, the measurement range was determined to be 0.65–120.00 ng/mL. The mean inter- and intra-assay variations were 12% and 4%, respectively, and linearity showed acceptable dilution recovery up to 50% dilution. The analyte was stable for up to five freeze–thaw cycles in human serum with 92–113% recovery. Hemoglobin, lipemia and biotin did not interfere with measurements of PRO-C9 in human serum. The human sequence was aligned with rodent sequences using UNIPROT (URL https://www.uniprot.org/align, accessed on 21 December 2023), and the corresponding sequence in mouse and rat had a mismatch on position 5 (Figure 2A). The mAb towards the selection peptide was shown to be specific, as no reactivity was shown towards the elongated, truncated or non-sense peptide (Figure 2B).

### 3.2. PRO-C9 Is Elevated in Patients with Various Types of Cancers Compared to Healthy Controls

The PRO-C9 assay was measured in sera from 11 groups of patients with different solid tumors and healthy donors. Each group included patients diagnosed with bladder (*n* = 19), breast (*n* = 20), colorectal (*n* = 20), gastric (*n* = 20), head and neck (*n* = 20), lung (*n* = 60), melanoma (*n* = 20), ovarian (*n* = 20), pancreatic (*n* = 20), prostate (*n* = 20), or renal cancer (*n* = 20), together with healthy donors (*n* = 73) (Table 3).

**Figure 3:**
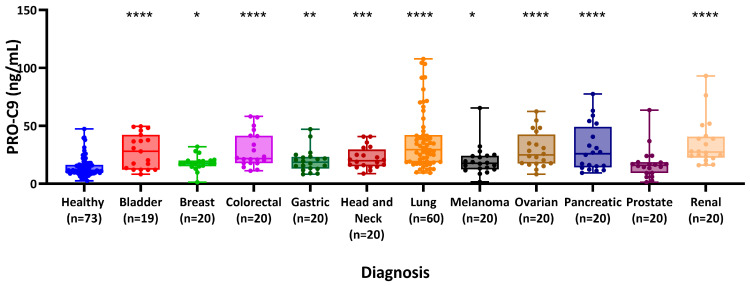
Serum levels of PRO-C9 were measured in a cohort comprising healthy donors (*n* = 73) and patients with various cancers including bladder (*n* = 19), breast (*n* = 20), colorectal (*n* = 20), gastric (*n* = 20), and head and neck (*n* = 20). Statistical significance is indicated as follows: **** *p* < 0.0001, *** *p* < 0.001, ** *p* < 0.01, * *p* < 0.05, comparing cancer patients to healthy donors. The diagnostic power of PRO-C9 for the patients suffering from the different types of cancer compared to healthy controls showed an AUROC between 0.58 and 0.86 (all *p* < 0.01, except prostate cancer, with *p* = 0.3, Table 4).

Median PRO-C9 levels were found to be significantly elevated in patients with bladder cancer compared to healthy donors (28.00 vs. 11.55 ng/mL, *p* < 0.0001), as well as in those with breast (17.80 vs. 11.55 ng/mL, *p* = 0.014), colorectal (21.80 vs. 11.55 ng/mL, *p* < 0.0001), gastric (18.60 vs. 11.55 ng/mL, *p* = 0.0081), head and neck (19.80 vs. 11.55 ng/mL, *p* = 0.0007), lung (29.48 vs 11.55 ng/mL, *p* < 0.0001), melanoma (17.75 vs. 11.55 ng/mL, *p* = 0.012), ovarian (24.85 vs. 11.55 ng/mL, *p* < 0.0001), pancreatic (25.95 vs. 11.55 ng/mL, *p* < 0.0001), and renal cancer (27.37 vs. 11.55 ng/mL, *p* < 0.0001) (Figure 3). No significant differences were observed between patients with prostate cancer and healthy donors (15.95 ng/mL vs. 11.55 ng/mL, *p* = 0.15). 

## 4. Discussion

In this study, we developed and characterized a competitive CLIA, named PRO-C9, for the detection of type IX collagen using a monoclonal antibody targeting the C-terminal domain of the α1-chain of type IX collagen. The main findings of this study were as follows: (1) PRO-C9 assay wastechnically robust; (2) PRO-C9 was measurable in human serum, (3) PRO-C9 was significantly elevated in patients with bladder, breast, colorectal, gastric, head and neck, lung, melanoma, ovarian, pancreatic, prostate and renal cancer compared to healthy donors, and (4) PRO-C9 showed AUROC values between 0.58 and 0.89 when separating patients with solid tumors from healthy donors, indicating its potential as a diagnostic biomarker. 

The monoclonal antibody showed no cross-reactivity to either the elongated or truncated epitope, suggesting high specificity towards the targeted sequence. Additionally, it displayed low cross-reactivity with rodent species, based on a mismatch at position 5 within the human epitope sequence between human, mouse and rat. 

To our knowledge, this is the first study to show that the C-terminal domain of the α1-chain of type IX collagen can be measured non-invasively in blood and with biological relevance in patients with different solid tumor types. In agreement with our results, a previous study by Chung et al. [29] indicated that COL9A1 single nucleotide peptides are associated with oral cancer, while Piotrowski et al. [29,30], by studying microarrays, indicated that COL9A1 was apparent in tumors compared to healthy controls. 

Alterations in the composition and organization of the ECM, including changes in FACIT expression, are frequently observed in various cancers. Some tumor types demonstrate a differential expression of FACIT collagens, compared to normal tissues, potentially contributing to tumor progression, invasion, and metastasis [19,20,31,32]. Unlike other FACIT collagens primarily involved in regulating collagen fibrillogenesis and maintaining tissue integrity, such as type XII and XIV collagen, type IX collagen exhibits a distinct localization within the ECM [33]. Type IX collagen is typically found at the interface between fibril-forming collagens and the surrounding matrix, where it plays a crucial role in regulating fibril diameter and spacing [22]. This unique localization suggests that type IX collagen may have distinct functional roles compared to other FACIT collagens, particularly in modulating the mechanical properties and structural organization of tissues. We showed that levels of PRO-C9 were elevated in patients with colon cancer and breast cancer, and previous studies have shown that type XII collagen is also upregulated at the invasive forefront of colon cancer [34] and promotes breast cancer metastasis [32]. While both type IX and type XXII collagen are involved in regulating collagen fibril organization, they differ in their tissue distribution, structure, and specific functions. Type IX collagen is predominantly associated with cartilage and contributes to its mechanical properties, whereas type XXII collagen has a broader tissue distribution and is implicated in various developmental processes beyond cartilage formation [35]. In a previous study involving the same cohort as our current research, a biomarker for type XXII collagen (PRO-C22) also exhibited elevated levels in patients with solid tumors compared to healthy controls. Additionally, in combination with PRO-C3, a marker for type III collagen formation associated with fibrosis, it demonstrated additive prognostic value [18]. Similar results were also found regarding a biomarker of type XX collagen (PRO-C20), where patients with solid tumors had elevated levels of PRO-C20 when compared to healthy controls [20]. In this later study, they also compared the FACIT collagen results with PRO-C1, measuring type I collagen formation, and found that PRO-C20 holds diagnostic potential, highlighting the potential of characterizing collagens of low abundance, such as FACITs.

These collective findings underscore the important role of FACITs in the context of cancer, even though additional studies need to be performed to understand the mechanisms of each of them in the development and progression of cancer. Understanding the distinct properties of type IX collagen and its implications in cancer biology may provide valuable insights into the development of diagnostic and therapeutic strategies targeting the ECM in cancer. However, the current exploratory study faced several limitations. The study was cross-sectional, and the small sample size may have introduced biases. Clinical information was very limited, with no data available on tumor size or the time from diagnosis to blood collection. To strengthen our findings, complementary studies on animal models should be conducted to quantify the type IX collagen biomarker. Additionally, immunohistochemistry analyses of type IX collagen in solid tumors should also be performed. These limitations highlight the importance of reproducing these findings in independent, prospective, and well-characterized studies.

## 5. Conclusions

In conclusion, our study introduced and successfully assessed the technical robustness of PRO-C9, a CLIA targeting the C-terminal of type IX collagen. PRO-C9 levels were quantified in sera from patients with different solid tumors, and we found them significantly elevated compared to healthy controls. These findings suggest that PRO-C9 could potentially serve as a cancer biomarker, after further assessment in well-characterized studies, underlying the importance of FACIT biology in cancer. Further research investigating the specific roles of FACITs in different aspects of cancer biology may uncover new opportunities for diagnosing and treating cancer more effectively.

## 6. Patents

Patent number of PRO-C9 biomarker: 2218042.6.

## Figures and Tables

**Table 1:** Sequences of the synthetic peptides used for monoclonal antibody production, assay development and validation.

Peptide Type	Sequence
Immunogenic peptide	KLH *-CGG-QRAFNKGPDP
Selection peptide	QRAFNKGPDP
Elongated selection peptide	QRAFNKGPDPG
Truncated selection peptide	QRAFNKGPD
Non-sense coating and standard peptide	DQAAGGLRQH

* Keyhole Limpet Hemocyanin.

**Table 2:** Summary of technical parameters for PRO-C9.

Assay Parameter	Result
ELISA format	Competitive CLIA
Curve fit model	4-point logistic curve fit
Detection range: LLOQ-ULOQ	0.65–120.00 (ng/mL)
LLOB	0.41 ng/mL
Mean Slope	1.022
Mean IC25	1.20 ng/mL
Mean IC50	3.62 ng/mL
Mean IC75	10.61 ng/mL
Inter-assay CV% (Range)	5.23–26.01
Intra-assay CV% (Range)	2.35–5.93
Dilution recovery (1 + 1, Range)	94.6–100.8%
Accepted maximum freeze–thaw cycles	Five freeze–thaw cycles (92–113%)
Interference hemoglobin, low/high	94%/102%
Interference lipid, low/high	103%/102%
Interference biotin	>100 ng/mL

Abbreviations: CLIA, chemiluminescence immune assay; LLOB, lower limit of blank.

**Table 3:** Patient demographics of clinical cohort.

	Cancer (*n* = 259)	Healthy (*n* = 73)
Sex, male, *n* (%)	152 (58.7%)	32 (43.8%)
Ethnicity		
- Missing		29
- Black		6 (13.6%)
- Caucasian	259 (100.0%)	28 (63.6%)
- Hispanic		10 (22.7%)
Age, Mean (SD)	59.4 (10.7)	48.2 (9.3)
Diagnosis		
- bladder cancer	19 (7.3%)	
- breast cancer	20 (7.7%)	
- colorectal cancer	20 (7.7%)	
- head and neck cancer	20 (7.7%)	
- kidney cancer	20 (7.7%)	
- lung cancer	60 (23.2%)	
- melanoma	20 (7.7%)	
- ovarian cancer	20 (7.7%)	
- pancreatic cancer	20 (7.7%)	
- prostate cancer	20 (7.7%)	
- renal cancer	20 (7.7%)	

Abbreviations: SD, standard deviation.

**Table 4:** ROC curve analysis was applied to evaluate the ability of PRO-C9 to distinguish between healthy controls and cancer patients.

Diagnosis	AUC	Threshold	Sensitivity	Specificity	ppv	npv	*p*-Value
Bladder	0.79 [0.67–0.90]	11.9	0.95	0.53	0.35	0.97	0.0001
Breast	0.73 [0.61–0.85]	14.7	0.85	0.63	0.39	0.94	0.002
Colorectal	0.84 [0.76–0.93]	17.1	0.85	0.77	0.5	0.95	<0.0001
Gastric	0.70 [0.57–0.83]	11.8	0.85	0.53	0.33	0.93	0.006
Head and neck	0.78 [0.67–0.88]	14.2	0.90	0.62	0.39	0.96	0.0002
Kidney	0.90 [0.85–0.96]	16.0	1.00	0.75	0.53	1.00	<0.0001
Lung	0.86 [0.80–0.92]	16.1	0.87	0.75	0.74	0.87	0. 0001
Melanoma	0.69 [0.56–0.82]	15.7	0.65	0.71	0.38	0.88	0.008
Ovarian	0.83 [0.73–0.93]	16.6	0.85	0.77	0.5	0.95	<0.0001
Pancreatic	0.79 [0.68–0.90]	24.9	0.55	0.89	0.58	0.88	<0.0001
Prostate	0.58 [0.42–0.74]	13.1	0.70	0.58	0.31	0.88	0.3

Abbreviations: AUC, area under the curve; ppv, positive predictive value; npv, negative predictive value.

## Data Availability

The data presented in this study are available upon reasonable request from the corresponding author.
